# Short-Term Effectiveness of a Lifestyle Intervention Program for Reducing Selected Chronic Disease Risk Factors in Individuals Living in Rural Appalachia: A Pilot Cohort Study

**DOI:** 10.1155/2014/798184

**Published:** 2014-01-16

**Authors:** David Drozek, Hans Diehl, Masato Nakazawa, Tom Kostohryz, Darren Morton, Jay H. Shubrook

**Affiliations:** ^1^Department of Specialty Medicine, Ohio University Heritage College of Osteopathic Medicine, Athens, OH 45701, USA; ^2^School of Medicine, Loma Linda University, Loma Linda, CA 92354, USA; ^3^Office of Research and Grants, Ohio University Heritage College of Osteopathic Medicine, Athens, OH 45701, USA; ^4^Live Healthy Appalachia, Athens, OH 45701, USA; ^5^Lifestyle Research Center, Avondale College of Higher Learning, Cooranbong, NSW 2265, Australia; ^6^Department of Family Medicine, Ohio University Heritage College of Osteopathic Medicine, Athens, OH 45701, USA

## Abstract

Most Western chronic diseases are closely tied to lifestyle behaviors, and many are preventable. Despite the well-distributed knowledge of these detrimental behaviors, effective efforts in disease prevention have been lacking. Many of these chronic diseases are related to obesity and type 2 diabetes, which have doubled in incidence during the last 35 years. The Complete Health Improvement Program (CHIP) is a community-based, comprehensive lifestyle modification approach to health that has shown success in addressing this problem. This pilot study demonstrates the effectiveness of CHIP in an underserved, rural, and vulnerable Appalachian population. Two hundred fourteen participants in CHIP collectively demonstrated significant reductions in body mass index, systolic and diastolic blood pressure, and fasting blood levels of total cholesterol, low-density lipoprotein, and glucose. If these results can be repeated in other at-risk populations, CHIP has the potential to help reduce the burden of preventable and treatable chronic diseases efficiently and cost-effectively.

## 1. Introduction

Chronic diseases are on the rise, accounting for 84% of the current national healthcare expenditure [1]. Nearly 18% of the US Gross Domestic Product, or $2.7 trillion, is now being spent on healthcare. This is by far more than any other nation [2]. Many of these chronic diseases have lifestyle underpinnings and are responsive to lifestyle modification [3]. It is estimated that nearly 40% of all cancer deaths and 82% of cardiac deaths could be prevented. In addition, 71% of colon cancer, 71% of strokes, and 91% of the cases of diabetes could be avoided through appropriate lifestyle changes by adopting a simpler, healthier diet, by following a consistent activity program and by avoiding tobacco [4].

The Complete Health Improvement Program (CHIP) [5] is an intensive community-based lifestyle intervention that has been shown to offer significant benefits for the prevention, control, and even reversal of cardiovascular disease [6–8], type 2 diabetes (T2DM) [9, 10], and depression [11, 12]. This program is largely attractive to middle-class people who are generally employed, have the means to enroll, and have a level of education that facilitates the understanding, assimilation, and application of the healthy lifestyle principles presented in the program.

In Appalachia, Athens County is struggling with the highest poverty level in Ohio at 35% [13], with nearly 18% of the population uninsured [14]. Many people in this region are struggling with issues related to poverty, such as limited access to health care, inadequate housing and transportation, and limited education. Would these challenges hinder their ability to afford, attend, assimilate, and apply the behavioral changes taught in CHIP? To address this issue, several CHIP classes aimed at the general Athens population were conducted. How would the general population fare when compared to other CHIP classes across America? And if the results were comparable, could strategies be developed that could reach those in the underserved and marginalized lower socioeconomic groups in Appalachia? The aim of this pilot study, then, is to examine the short-term effectiveness of the CHIP intervention in the *general *population of Athens County, Ohio, in Appalachia, for reducing selected chronic disease risk factors.

## 2. Methods

The study examined the changes in selected chronic disease risk factors of 225 self-selected participants who attended one of six CHIP classes offered in 2011 and 2012 in Athens, Ohio. Approval for the study was obtained from the local CHIP administration and the Ohio University Institutional Review Board.

### 2.1. Description of CHIP

The CHIP classes were facilitated by volunteers trained and authorized by the Lifestyle Medicine Institute/CHIP through Athens CHIP and were administered locally by Live Healthy Appalachia (LHA), a 501(c)3 organization, located in Athens, Ohio. Each class was conducted over a 4- to 8-week period and involved 16 two-hour group sessions. A typical session included viewing an instructional video, a cooking demonstration, group discussion, and an exercise component. The intent of the intervention was to nurture intelligent self-care through enhanced understanding of the epidemiology, etiology, and risk factors associated with chronic western diseases. The cost of the course covered program tuition, two biomedical assessments (performed at the beginning and near the end of the class), food samples, textbook, workbook, cookbook, water bottle, pedometer, and supplementary reading and reference material.

The primary focus of CHIP was the consumption of whole foods* ad libitum*, such as fresh fruits, vegetables, whole grains, legumes, and some nuts. The goal was to keep overall dietary fat content below 20% of the total calories and the daily intake of added sugar below 10 teaspoons, sodium below 2,000 mg, and cholesterol below 50 mg. Water consumption (at least 8 glasses/day) and high fiber food intake (>35 g/day) were encouraged, along with flexibility exercises and a daily walk of 30 minutes, with a goal of reaching 2 miles or 10,000 steps on the pedometer.

Although most classes were conducted in 4 weeks, meeting 4 times a week, a couple of classes were extended to 8 weeks, with fewer meetings per week. The course tuition increased incrementally over the time frame covered by the study from $350 to $450. The cost was paid by the participants, by their employer, by a scholarship, or by any combination of these. A limited number of scholarships (for 22 of the 225 participants) were provided by local community organizations and businesses, earmarked for participants with financial need. Scholarship recipients were selected by the local CHIP administration based on interest, expression of need, and eligibility for financial support.

An initial health screen was performed at the beginning of the course. The results were reviewed with the participants to help them understand their risk status and to set goals for the program.

After the second health screen, personal and deidentified aggregated class health screen results were given to each participant to see their individual improvements and how they compared to the group as a whole. This was accompanied by a presentation on the meaning of the results and encouragement to continue with the newly acquired lifestyle changes. Additional copies of results were provided along with encouragement to share these with their primary care provider.

### 2.2. Study Participants

Study participants were self-selected, learning about CHIP via announcements in churches and the local media, or from local health care providers. Potential CHIP participants attended one of several informational sessions presented throughout the community on various dates and times, where they received a mixture of video and live presentations, had their questions addressed, and were offered an opportunity to enroll. All participants in CHIP were informed that their results would be aggregated and reported for research purposes. They were given the option of having their data excluded without affecting their eligibility to participate in CHIP. As shown in Table 3, this cohort at baseline was representative of an at-risk population with values high in body mass index (BMI), blood pressure, fasting lipids, and glucose.

### 2.3. Data Collection and Reporting

The biomedical assessments included weight, height, systolic blood pressure (SBP), and diastolic blood pressure (DBP), obtained by trained medical professionals. Fasting blood samples were collected by trained phlebotomists and analyzed for total cholesterol (TC), low-density lipoprotein (LDL), high-density lipoprotein (HDL), triglycerides (TG), and plasma glucose (FPG) utilizing a Beckman Coulter DXC-600 analyzer in the pathology laboratory at O'Bleness Memorial Hospital in Athens, Ohio, a lab certified by the American College of Pathologists,.

Data for each participant was entered into a password protected proprietary Access-based database maintained on the CHIP administration computer at the LHA office as part of the CHIP routine and separate from the data collection for this study. For this study, CHIP administration provided aggregated data from the first six CHIP classes, without personal identifiers, on a password-protected Excel database file.

### 2.4. Data Analysis

For both overall and stratified data, means and standard deviations (SD) were computed for each baseline and postintervention. Mean change (baseline mean − postintervention mean) and percent (%) mean change (100 × mean change/baseline mean) were also computed to show magnitudes of changes. One-sample *t*-tests were applied to the % mean changes to test whether these changes were significant. Cohen's *d* was computed within each stratum to show effect size. McNemar tests were also utilized to examine whether frequency distributions of participants across the risk factor strata changed from baseline to postintervention. For a reference purpose, two-sample *t*-tests were used to compare the magnitude of changes between Athens CHIP and Rankin et al. [8]. Rankin et al. evaluated the results of CHIP classes throughout multiple sites in the US, excluding Athens.

## 3. Results

In this pilot study, a total of 225 people enrolled, with a mean age of 56 years and with a range of 24–81 years, 28% male, 72% females, as displayed in Table 1. Of those enrolled, 210 (90%) participated in all aspects of the study; 214 (95%) completed the first and at least part of the second health screen. The mean difference “before” and “after” biometric value is represented in Table 2. The participants achieved significant mean clinical changes in almost all risk factors, as demonstrated by the *P* values.

Table 2 also displays the mean changes of 5066 participants who attended CHIP classes throughout the US, excluding those attending the Athens classes, as reported by Rankin et al. [8]. The findings in Athens were comparable to those seen in Rankin et al., as demonstrated by the *P*: (1) versus (2).

Table 3 displays the *stratified* data using conventional risk factor categories. The data in all substrata improved in all risk factors except those in the normal range for SBP, DBP, and TG, as demonstrated by the *P* values and Cohen's *d*. Please note that the higher the risk strata when entering the program, the greater the improvements by the end of the intervention.

A comparison of the risk factor reductions observed in this study with those reported by Rankin et al. from CHIP chapters across the United States was similar, as was the data presented in Table 1 looking at gender and age differences among those two groups. This was the case even though the Athens classes attracted a wider age spread.

## 4. Discussion

The aim of this study was to validate the effectiveness of CHIP in the rural Appalachian setting of Athens County, Ohio. A total of 225 self-selected participants had the financial resources available to attend the 4- to 8-week intensive, community-based lifestyle intervention program. Chronic disease-related risk factors responded favorably and rapidly to the recommended lifestyle changes towards a healthier whole food, plant-based diet coupled with daily exercise. Those at highest risk had the greatest improvements. The substantial changes in BMI, especially among those in the obesity category, are remarkable in that the program emphasized the unrestricted use of whole foods (fruits, vegetables, whole grains, and legumes) plus plenty of water (to replace carbonated drinks and caffeine). The emphasis was on an *ad libitum* diet without restrictions on serving size or the amount of food eaten. The recommended daily exercise burned insufficient calories to explain the amount of weight loss that occurred. Unlike calorie-restrictive diets, which leave the participant feeling hungry, the low-caloric density of food high in fiber filled the stomach, providing satiation naturally, and contributed to the success of the program.

These findings follow those from the Diabetes Prevention Program (DPP), which documented the efficacy of a lifestyle program in those at risk for diabetes [15]. Those who were in the lifestyle arm of the study had a 58% reduction in the diagnosis of T2DM, far better than the pharmacologic arm using metformin (31%). The Look AHEAD trial evaluated a lifestyle intervention for those diagnosed with T2DM [16]. The goals of this program were to attain >7% body weight loss through a calorie-restricted diet (1200–1800 daily calories, <30% calories from fat) and a gradual increase in activity to 175 minutes per week. The lifestyle intervention group succeeded in losing weight and improving diabetes risk factors (SBP, DBP, TG, and hemoglobin A1c). These two studies are in line with results achieved in the Rockford CHIP project [10]. In this cohort of 84 people with diabetes who were on oral medication, 38 dropped their mean FPG by 18%. This improvement occurred even as medication dosage was reduced by more than 10%. Similarly, of 46 participants who were on insulin, 18 were able to drop their mean FPG by 22% while at the same time reducing insulin dosage by more than 10% [10].

The overall clinical changes in this pilot study are similar to those found in other 4-week CHIP classes throughout United States [6–8]. Other groups also have documented large serum cholesterol drops in response to a plant-based eating pattern [17, 18]. The emphasis on substantially reducing dietary cholesterol, as well as saturated and trans fat, while increasing dietary fiber, may largely explain significant drops in elevated TC and LDL levels.

The significant reductions in TC and LDL may possibly explain the reduction in HDL levels, which is usually considered detrimental. On the other hand, a systematic review and meta-analysis has questioned this. Evaluating 108 randomized trials involving close to 300,000 participants at risk for coronary artery disease (CAD), an international review group concluded that “simply increasing the amount of circulating HDL does not reduce the risk of CAD events, CAD deaths, or total deaths [19].” More recently, views are emerging that HDL may have both atheroprotective as well as proinflammatory/atherogenic properties [20–22]. Could it be that whole food, plant-based diets that have successfully demonstrated the regression of CAD in response to markedly lower lipid values, including HDL, may have facilitated the conversion of HDL from a proinflammatory to an anti-inflammatory, atheroprotective particle [23–25]? And could it be that these properties are more important than the absolute HDL numbers [26, 27]?

Several groups have demonstrated CAD regression [23, 24, 27–29] by utilizing a lifestyle medicine approach centered on a simpler diet consisting of more plant-based whole foods [23, 29]. Other researchers achieved similar results, having included daily exercise in the program [17, 24, 29]. It not only lowers elevated blood pressure, but it also lowers blood glucose levels and the corresponding dosage of needed medication. At the same time, excess weight levels decrease as participants eat, without restriction of quantity, more foods as grown, simply prepared.

According to other studies, concurrently with improvement in biomarkers and improved health, the CHIP lifestyle intervention program is cost-effective, reducing medical costs for participants [30–32]. A New York Academy of Medicine review of community prevention programs that are proven to be effective, including CHIP, estimated that they produce a return on investment (ROI) of 5.6 over five years [33].

Despite the evidence of effectiveness, lifestyle medical treatment is generally underutilized. The prevalent conventional one-to-one, provider-to-patient health care model is significantly limited in the following ways. (1) The time allocated per patient is short, averaging less than 15 minutes, and usually spent addressing problems and prescribing or renewing a different medication for each problem. (2) The model incentivizes the physician to choose the quickest and least time-intensive, but often the most expensive method of evaluation and treatment for the patient. (3) The model is expensive in that it requires highly trained and therefore highly compensated providers to provide basic information that could easily be taught in a more efficient, less expensive way. (4) The model is driven by illness, not by wellness, compensating providers for addressing problems, not for maintaining health. (5) Finally, in many localities, there is a shortage of primary care providers, leaving many who are in need without a medical home to address prevention and easily managed health issues. Left untended, these can then escalate into serious problems, leading the patient to seek care via emergency services, greatly elevating the cost of care.

CHIP, though not generally reimbursed by insurers, historically has utilized trained volunteer facilitators. The facilitators, who are not necessarily medical professionals, present a prepared curriculum in a group setting, achieving remarkable results in reducing biomarkers for illness, medication consumption, and overall health care costs. The content in the videos and printed materials, as well as the feedback from the initial health risk assessment, has been demonstrated to be the key to the success of CHIP. The results of CHIP are less dependent on the training and professional qualification of the facilitator. CHIP can therefore be presented more efficiently and cost-effectively by lower-paid, nonmedical, nonprofessional facilitators in a group setting. It is the final health risk assessment and its consistently favorable clinical outcomes, often accompanied by reductions in medication requirements, as well as the understanding that the participants have become the major contributor to their own health, which provides the needed motivation to maintain the new lifestyle and its health-related benefits.

### 4.1. Limitations

The magnitude of the observed changes in the biometric risk factors may relate, in part, to the self-selection of the participants. These were people who could afford or obtain funding for the program and thus demonstrated their readiness for a change to improve their health and to assume more personal responsibility for behavioral adjustments, especially in their daily diet. There may also be the confounder of *regression to the mean,* although a randomized controlled trial of the Rockford CHIP program discounted this as a major explanation for the magnitude of the observed favorable changes [34]. These are only short-term measures of behavioral changes. To what extent they can be maintained remains to be seen, since short-term behavior changes are subject to decay over time [35]. Future investigation needs to be targeted at the effectiveness of a maintenance program with regularly scheduled educational meetings and at the effect of heightened community awareness and interest, demonstrated by an increasing number of food suppliers and restaurants selling and serving plant-based whole foods and CHIP menu selections in the region.

## 5. Conclusion

Athens County is an economically struggling area with the highest poverty rate in Ohio at 35%, with nearly 18% of the population uninsured. To address this need, in 2010, Live Healthy Appalachia became a regional provider of CHIP, attempting to bring better health through education, motivation, and inspiration. This retroactive analysis assessing the cumulative effects of six CHIP classes conducted in the Athens area by LHA has shown that self-selected participants can respond and achieve clinical improvements not dissimilar to those achieved from other sites across the US.

But, can this program be applied to the many people living in poverty in Appalachia with significant health issues, often lacking transportation, adequate education, and easy access to healthcare? Further innovative methods for presentation and application of the principles taught in CHIP may need to be developed and assessed, which will address the needs of people whose situations may hinder their ability to afford, attend, assimilate, and apply the behavioral changes currently taught in CHIP.

## Figures and Tables

**Table 1:** Comparison of gender and mean age distribution in participants attending the Athens, OH, CHIP classes versus those who attended CHIP classes in the US, excluding those attending the Athens classes (Rankin et al. [8]).

Characteristics	Athens	Rankin
Total	225	5,066
Male	63	1,694
Female	162	3,372
% male	28	33
% female	72	67
Age: mean (range)	56.0 (24–81)	52.7 (44–71)

**Table 2:** Mean changes in selected risk factors in 225 participants of the Athens, OH, CHIP classes, compared to 5066 participants who participated in CHIP interventions in the US (Rankin et al. [8]).

Variables	Athens (1)				Rankin study (2)				*P*: (1) versus (2)
	*n*	Δ (%)	SD	*P*	*n*	Δ (%)	SD	*P*	
BMI (kg/m^2^)	208	−3.5	4.0	<0.001	4536	−3.2	3.0	<0.001	>0.2
SBP (mmHg)	203	−4.7	11.5	<0.001	4579	−4.9	18.7	<0.001	>0.2
DBP (mmHg)	203	−1.7	13.6	0.085	4577	−5.3	12.9	<0.001	<0.001
TC (mg/dL)	214	−10.3	13.2	<0.001	4674	−11.0	13.8	<0.001	>0.2
LDL (mg/dL)	210	−10.3	21.5	<0.001	4568	−13.0	21.1	<0.001	0.080
HDL (mg/dL)	214	−10.2	13.0	<0.001	4673	−4.7	8.8	<0.001	<0.001
TG (mg/dL)	214	0.0	38.0	>0.2	4669	−7.7	42.8	<0.001	0.005
FPG (mg/dL)	213	−4.1	11.1	<0.001	3689	−6.1	21.3	<0.001	0.018

**Table:** (a)

Risk factor	*N*	*N*	*χ* ^2^* (*P*)	Baseline		Post-intervention		Δ	Δ	*P*	Cohen's *d*
	Baseline	Post-intervention		Mean SD		Mean SD		Mean	%		
BMI (kg/m^2^)											
18.5–24.9	35	51	31 (<0.001)	22.7	1.7	22.3	2.6	−0.4	−1.8%	>0.2	0.20
25–30	57	58		27.3	1.3	26.1	1.4	−1.2	−4.4%	<0.001	1.90
>30	116	99		37.6	6.4	36.2	6.2	−1.4	−3.7%	<0.001	1.51
SBP (mmHg)											
<120	66	104	38 (<0.001)	111.3	8.3	113.0	10.9	1.7	1.5%	0.169	−0.17
120–139	91	78		130.3	5.6	122.7	12.1	−7.6	−5.8%	<0.001	0.64
140–160	38	19		148.8	5.6	130.4	14.4	−18.4	−12.4%	<0.001	1.29
>160	8	2		170.4	10.5	150.2	21.1	−20.2	−11.9%	0.01	1.24
DBP (mmHg)											
<80	130	154	12 (<0.001)	70.8	6.6	71.9	8.3	1.1	1.6%	0.066	−0.16
80–89	54	39		85.0	3.3	78.9	8.1	−6.1	−7.2%	<0.001	0.74
>90	19	10		94.4	2.6	82.4	8.9	−12	−12.7%	<0.001	1.39
TC (mg/dL)											
<160	51	108	59 (<0.001)	141.5	15.1	133.1	27.7	−8.4	−5.9%	0.009	0.38
160–199	97	63		181.2	11.7	160.8	22.1	−20.4	−11.3%	<0.001	1.02
200–239	47	35		218.3	10.8	189.2	26.7	−29.1	−13.3%	<0.001	1.07
240–280	16	7		253.2	10.0	233.2	25.1	−20	−7.9%	0.015	0.69
>280	3	1		303	29.7	216.3	55.7	−86.7	−28.6%	0.192	1.12

**Table:** (b)

Risk factor	*N*	*N*	*χ* ^2^* (*P*)	Baseline		Post-intervention		Δ	Δ	P	Cohen's *d*
	Baseline	Post-intervention		Mean SD		Mean SD		Mean	%		
LDL (mg/dL)											
<100	78	115	52 (<0.001)	78.3	18.6	72.9	23.5	−5.4	−6.9%	0.114	0.18
100–129	67	56		115.0	8.2	99.5	18.3	−15.5	−13.5%	<0.001	0.86
130–159	46	33		143.1	9.8	125.7	19.8	−17.4	−12.2%	<0.001	0.94
160–190	14	6		169.4	7.0	145.6	25.1	−23.8	−14.0%	0.004	0.92
>190	5	0		210.6	19.2	141.4	29.1	−69.2	−32.9%	0.015	1.83
HDL (mg/dL)											
<40	69	105	60 (<0.001)	34.9	3.7	33.4	4.6	−1.5	−4.3%	0.004	0.36
40–60	101	89		48.8	5.6	42.2	7.0	−6.6	−13.5%	<0.001	1.16
>60	44	20		72.1	13.7	62.5	15.7	−9.6	−13.3%	<0.001	0.86
TG (mg/dL)											
<100	80	84	1.5 (>0.2)	71.3	19.0	81.3	32.8	10.0	14.0%	0.001	−0.38
100–199	102	103		143.7	29.7	133.5	43.5	−10.2	−7.1%	0.023	0.23
200–400	29	26		256.3	43.0	206.5	84.5	−49.8	−19.4%	0.002	0.63
>400	3	1		444.7	57.6	219.0	48.9	−225.7	−50.8%	0.019	4.16
FPG (mg/dL)											
<110	162	181	15 (<0.001)	94.7	8.8	93.2	9.1	−1.5	−1.6%	0.021	0.18
110–125	27	18		116.2	4.0	108.1	11.9	−8.1	−7.0%	<0.001	0.70
>125	24	14		169.1	52.3	131.6	29.9	−37.5	−22.2%	<0.001	0.99
